# Immunohistochemical Expression of p62 in Feline Mammary Carcinoma and Non-Neoplastic Mammary Tissue

**DOI:** 10.3390/ani12151964

**Published:** 2022-08-02

**Authors:** Gian Enrico Magi, Francesca Mariotti, Lorenzo Pallotta, Alessandro Di Cerbo, Franco Maria Venanzi

**Affiliations:** 1 School of Biosciences and Veterinary Medicine, University of Camerino, 62024 Matelica, Italy; gianenrico.magi@unicam.it (G.E.M.); lorenzo.pallotta@studenti.unicam.it (L.P.); alessandro.dicerbo@unicam.it (A.D.C.); 2 CureLab Oncology Inc., Dedham, MA 02026, USA; francovenanzi51@gmail.com

**Keywords:** p62, cat, mammary gland, carcinoma, autophagy

## Abstract

**Simple Summary:**

The p62 protein, also called sequestosome 1 (SQSTM1), is a ubiquitin-binding scaffold protein. The expression of p62 was statistically higher in carcinoma compared to non-neoplastic mammary glands. Our results, although preliminary, are similar to the results of breast cancer, therefore, also in the cat, p62 could be considered a possible oncotarget.

**Abstract:**

The p62 protein, also called sequestosome 1 (SQSTM1), is a ubiquitin-binding scaffold protein. In human oncology, although the interest in the function of this protein is recent, the knowledge is now numerous, but its role in tumorigenesis is not yet clear. This preliminary study aims to evaluate the immunohistochemical expression of p62 in 38 cases of feline mammary carcinoma with different grades of differentiation and in 12 non-neoplastic mammary gland tissues, to assess the expression level and a possible correlation with malignancy. The expression of p62 was statistically higher in carcinoma compared to non-neoplastic mammary glands: 28 feline mammary carcinomas (73.7%) had a high p62 expression score, three (7.9%) had a moderate expression, while seven cases (18.4%) had a low expression. The grade of the differentiation of the carcinoma was not correlated with the p62 expression. This study represents the first approach in feline oncology that correlates p62 expression in feline mammary carcinoma. Our results, although preliminary, are similar to the results of human breast cancer, therefore, also in the cat, p62 could be considered a possible oncotarget.

## 1. Introduction

The p62 protein, also called sequestosome 1 (SQSTM1), is a “scaffold” protein (capable of bringing other proteins together), fundamental in the process of autophagy, linked to ubiquitin, which polymerizes through an N-terminal PB1 domain and which can interact with ubiquitinated proteins through the C-terminal UBA domain [1]. p62 is found in cellular inclusion bodies together with polyubiquitinated proteins and in cytosolic protein aggregates that accumulate in the course of various chronic degenerative diseases such as in Lewy bodies in Parkinson’s disease, in neurofibrillary clusters of Alzheimer’s disease, in Paget’s bone disease, and the Huntingtin aggregates in Huntington’s disease [2,3,4].

In human oncology, the cellular mechanism of autophagy has only recently been studied to understand its possible involvement in the carcinogenetic process. Autophagy is a non-selective mechanism, responsible for the degradation of the majority of long-lived cellular proteins and cell organelles [5]. Various studies have shown that both apoptosis and autophagy are pivotal factors for tumorigenesis, representing checkpoints for cell death and cell survival [6,7,8].

The metabolism of cancer cells is very different from that of normal cells: the high rate of cell proliferation, the increase in cell size, and the simpler enzymatic mechanisms require much oxygen and nutrients with the consequent production of high quantities of toxic compounds. Cancer cells are therefore able to reprogram their metabolism and activate the autophagy process in the various stages of the tumor growth [9].

Although the interest regarding the function of p62 is quite recent, in human oncology, several studies have been published. Nevertheless, the role of p62 in tumorigenesis is not very clear. p62 is considered a protein whose function arises at the crossroads between autophagy and apoptosis [10,11,12], although some authors ascribed to it a key role in autophagy-independent tumorigenesis. The results regarding the expression of p62 in various human cancers are heterogeneous, in particular, a high expression of p62 was found in the squamous cell carcinoma of the head and neck [13], prostate tumors [14,15], and different types of tumors of the digestive system [16]. In thyroid carcinomas, the expression of p62 changes in the different histotypes, resulting higher in the papillary type than in the follicular one [17]. In melanoma, p62 is considered a prognostic marker for the stage II [7], while esophageal adenocarcinoma with low p62 expression (both nuclear and cytoplasmic) has a worse prognosis [18]. Moreover, a study on colorectal tumors clearly demonstrated a correlation between the cytoplasmic localization of p62 and a favorable prognosis [8]. In human breast cancer, p62 is overexpressed in malignant cells [19]. The overexpression of p62 is correlated with tumor progression in triple-negative breast cancer and ovarian cancer [20].

In veterinary medicine, knowledge of the role of p62 in tumors is still limited, therefore, the purpose of this study is to evaluate the immunohistochemical expression of p62 in feline mammary carcinoma with different degrees of differentiation and in non-neoplastic mammary glands to highlight how this protein is expressed and if there is a correlation with the degree of the malignancy of the tumor. Mammary neoplasia in cats is less common compared to dogs, but when it occurs, most of the cases are malignant and histologic grading has a prognostic value [21].

## 2. Materials and Methods

### 2.1. Cases Included in the Study and Feline Mammary Carcinoma Grading System

This study included fifty cases: 12 post-mortem non-neoplastic mammary glands and 38 mammary carcinomas, of which 34 of the simple type and four of the ductal type were collected from 2012 to 2021 at the Pathology Unit of the School of Biosciences and Veterinary Medicine of the University of Camerino (Italy). From the same archive came reports and anamnesis of the cases. Ages ranged from 4 to 16 years, with a median age of 11.4 years, and all were European shorthair females. The specimens were fixed in 10% neutral buffered formalin, subsequently processed by an automatic histoprocessor, and embedded in paraffin. Serial sections (3 µm) were stained with hematoxylin and eosin (HE).

All feline mammary carcinoma cases included in the study underwent a histological grading system, following the more recent system proposed by Mills et al. [22] based on the Elston and Ellis system of female breast carcinoma, which assesses the presence of vascular invasion, the nuclear morphology and the mitotic count. The grading was performed considering an area of the tumor of 2.37 mm^2^. To each tumor, a score from 0 to 3 was assigned, and thus they were subsequently classified as grade I or low grade (score of 0), grade II, or intermediate cancers (score of 1), and grade III or higher cancers (score of 2–3).

### 2.2. Immunohistochemical Analysis and Immunoreactivity Scoring System for p62

Immunohistochemical analysis was carried out with a standard ABC-peroxidase method on 3 µm sections, as described by Di Cerbo et al. [23]. The labeling expression of p62 was evaluated using a rabbit polyclonal anti-p62 antibody (Sigma-Aldrich, St. Louis, MI, USA), diluted 1: 250.

As a secondary antibody, a goat anti-rabbit biotinylated antibody was used along with DAB (diaminobenzidine) as the chromogen. Normal mouse pancreas was used as a positive control [24], while rabbit IgG-isotype (instead of primary antibody) and a blocking peptide-based protocol were used as a negative control for both. Sections were counterstained in Mayer’s hematoxylin. Labeling was evaluated by two pathologists semi-quantitatively based on the percentage of immunopositive cells and staining intensity to obtain an expression score. An optical microscope (Leica DM 2500, Leica Microsystems Srl, Buccinasco, Italy) equipped with a camera (Leica DFC 7000T, Leica Microsystems Srl, Buccinasco, Italy) was used to acquire pictures. Each sample was firstly observed at 10 × magnification and then an area of the tumor of 2.37 mm^2^ was analyzed for the semiquantitative evaluation. A score was then assigned to each sample according to positive cell percentage and their signal intensity. The scoring system used was that described by Mariotti et al., 2019. [24]

Positive cell percentage was based on a 5-point scoring system: 0 = no positive cell, 1 = ≤20%, 2 = between 21% and 50%, 3 = between 51% and 70%, 4 = ≥71%. For each microscopic field, a count of immunopositive and negative cells was carried out and then transformed in percentage. The intensity of immunostaining was classified to a 4-point scoring system: 0 = no staining, 1 = low-intensity staining, 2 = moderate-intensity staining, 3 = high-intensity staining. In those samples where the intensity was heterogeneous, the score chosen was the predominant one in the sample itself. The overall score assigned to each case is derived by the multiplication of cell positivity and intensity signal score, with a minimum score of 0 and a maximum score of 12. The equivocal cases were evaluated by two authors (G.E.M. and F.M.) to establish the score. Scores between 1 and 2 were considered as a low expression of p62, those from 3 to 4 as intermediate, and those greater than 5 as high expression.

### 2.3. Statistical Analysis

Data were analyzed using GraphPad Prism 8 software (GraphPad Software, Inc., La Jolla, CA, USA). All data are presented as the means ± standard deviation (SD). Differences between the score of non-neoplastic patients and those with mammary carcinoma and differences between feline mammary carcinoma cases with lymphovascular invasion were analyzed using a Mann–Whitney test, while differences among different histological grades were analyzed using a Kruskal–Wallis’s test followed by Dunn’s multiple comparison test. The level of significance was set at * *p* < 0.05.

## 3. Results

**Figure 1:**
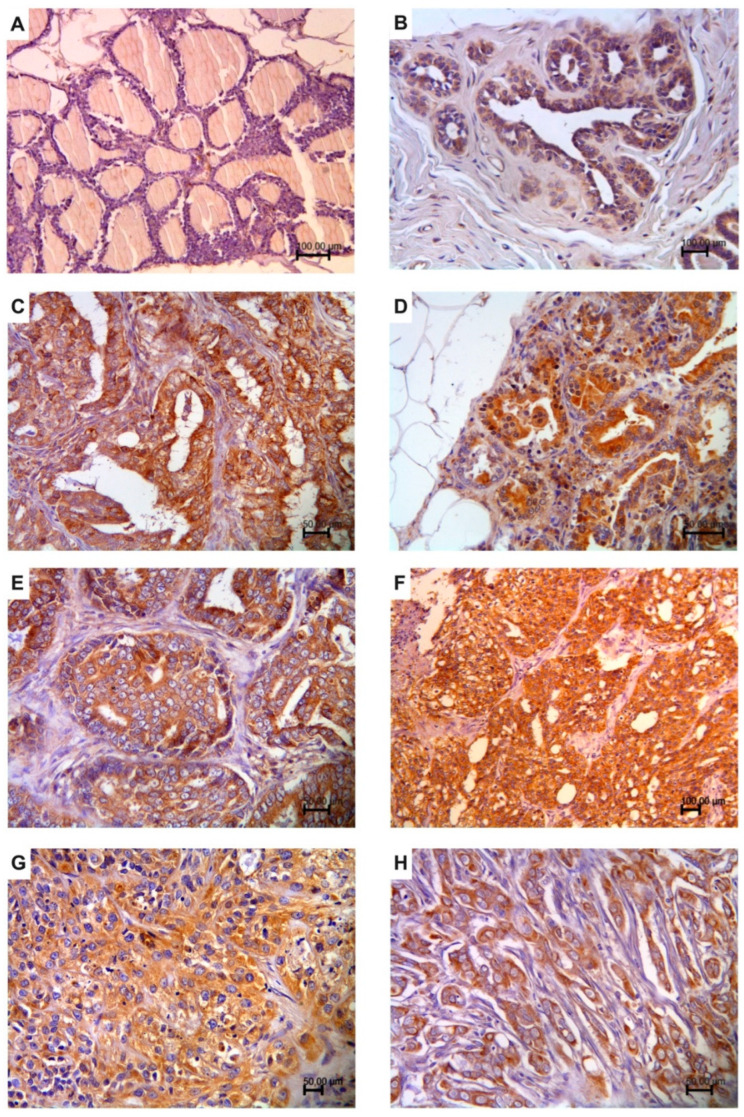
p62 immunostaining in the non-neoplastic feline mammary gland and mammary carcinoma (immunoperoxidase stain; hematoxylin counterstain). (**A**) Normal cat mammary gland negative for p62; (**B**) normal cat mammary gland slightly positive for p62; (**C**) low-grade (GI) feline mammary carcinoma with intense cytoplasmic immunolabeling; (**D**) low-grade GI feline mammary carcinoma with finely granular intense cytoplasmic immunolabeling; (**E**) intermediate-grade (GII) feline mammary carcinoma with intense cytoplasmic immunolabeling; (**F**) high-grade (GIII) feline mammary carcinoma with coarsely granular intense cytoplasmic immunolabeling; (**G**) high-grade (GIII) feline mammary carcinoma with intense granular cytoplasmic immunolabeling; (**H**) high-grade (GIII) feline mammary carcinoma with lymphovascular invasion and intense cytoplasmic immunolabeling.

Eight cases of non-neoplastic feline mammary gland tissue studied were negative for p62. Widespread negativity was observed in the epithelial cells of the mammary gland (Figure 1A); four cases out of twelve had some lobules with a slight positivity (Figure 1B). Positivity was never observed in the stromal cells.

The histological grading of the 38 cases of feline mammary carcinomas studied was as follows: five cases were low-grade GI (13.2%), 10 intermediate-grade GII (26.3%), and 23 high-grade GIII (60.5%).

**Figure 2:**
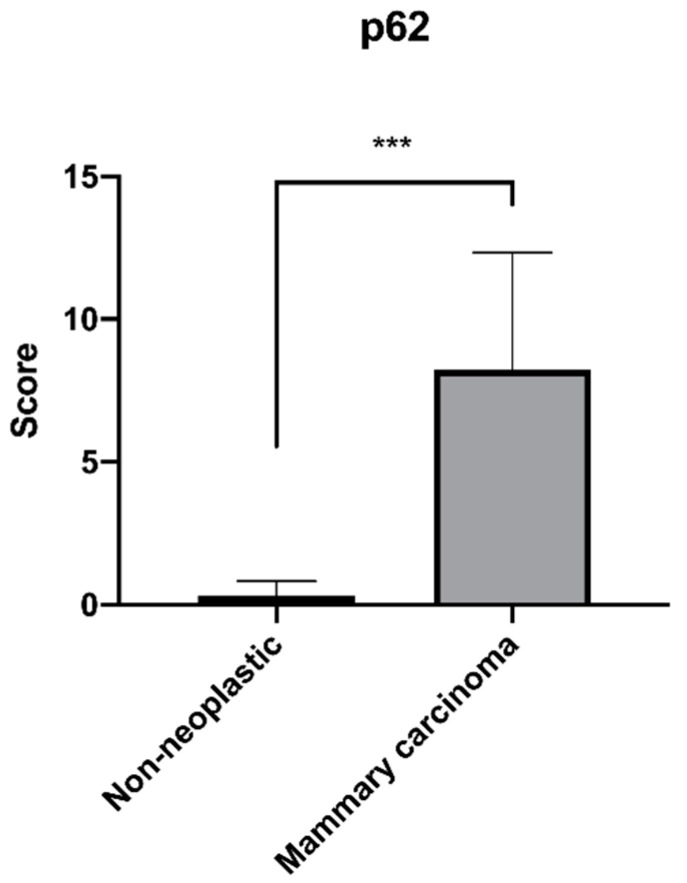
Mean (±SD) of the immunoreactivity score for p62 in the 38 cases of feline mammary carcinoma and 12 non-neoplastic tissue (*** *p* < 0.001).

Twenty-eight cases (73.7%) had a high p62 expression score (≥5), three (7.9%) had a score between 3 and 4, while seven cases (18.4%) had a low score between 1 and 2 (Table 1). Positive neoplastic cells showed cytoplasmic immunolabeling of varying intensity: mild to moderate to intense (Figure 1C–H). In some cases, the cytoplasmic immunolabel had a finely to coarsely granular appearance. Nuclear positivity was never observed in neoplastic epithelial cells and stromal cell positivity was not detected. The results of the p62 immunoreactivity scoring comparing non-neoplastic mammary glands and mammary carcinomas are shown in Figure 2. Between the two groups, there was a statistical difference in the score expression with a higher value in the mammary carcinoma group.

**Figure 3:**
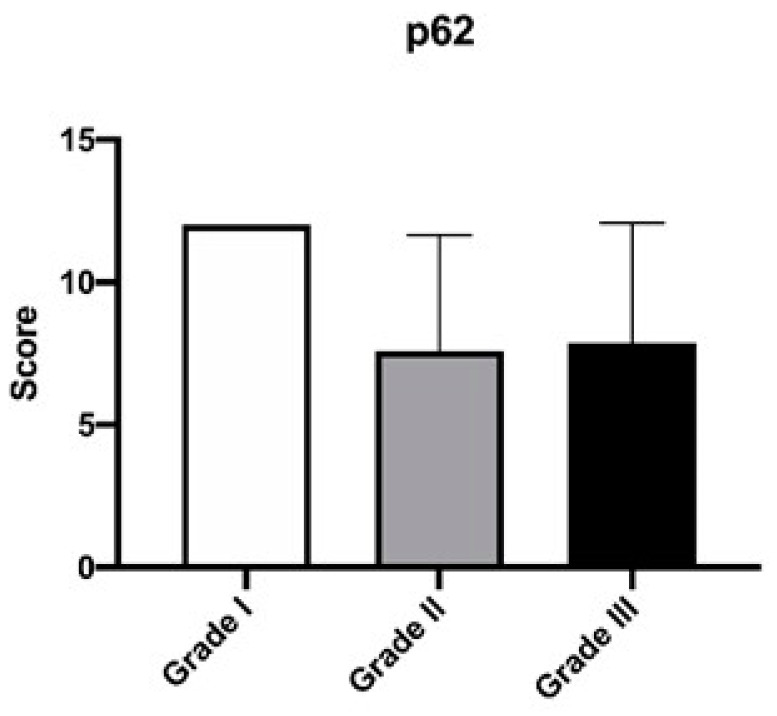
Mean (±SD) of the immunoreactivity score for p62 in the cases of feline mammary carcinoma with different grades of differentiation.

**Figure 4:**
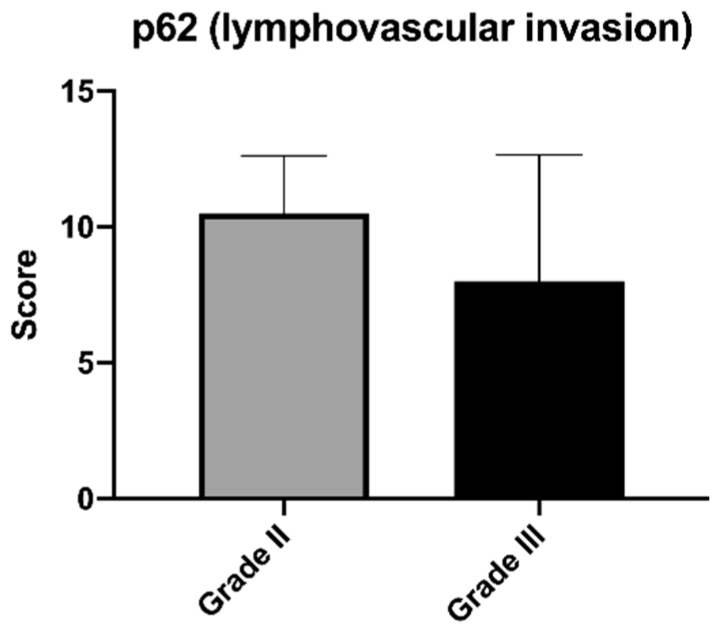
Mean (±SD) of the immunoreactivity score for p62 in the cases of feline mammary carcinoma with lymphovascular invasion considering the different grades of differentiation.

The correlation between the p62 expression score and the histological grade of the mammary carcinomas is reported in Table 1. We observed that five out of the twenty-eight cases with high p62 scores were low-grade, constituting 100% of all low-grade carcinomas studied, seven cases were intermediate-grade carcinomas (70%, seven out of ten), while for high-grade carcinomas, 16 out of the 23 cases studied had a high expression of p62 (69.6%). Among the three grades, there were no statistically significant differences in terms of p62 score expression (Figure 3). There were 10 cases with lymphovascular invasion (evidenced by the presence of neoplastic emboli within the vessels) (Figure 4), of which eight had a high histological grade (GIII), while two had an intermediate grade (GII). Seven out of ten cases (70%) were highly positive for p62 (Table 2).

## 4. Discussion

This preliminary study aimed to evaluate the immunohistochemical expression of the p62 protein in feline mammary carcinoma and feline mammary non-neoplastic tissue, taking into consideration tumors of different histological grades with different prognoses.

In this study, we observed a high positivity of feline mammary carcinoma for p62. These preliminary observations could suggest a correlation between the expression of p62 and the carcinogenic process since in carcinomas there is a high expression of the protein as opposed to non-neoplastic mammary gland tissue. Although is not possible to draw a definitive conclusion about the expression of p62 in feline mammary carcinomas, the statistical difference observed between mammary carcinoma and non-neoplastic tissue is a result to be considered.

In this study, no correlation was found between the expression of p62 and the different grades of mammary carcinoma, but the data obtained seem to show a certain tendency towards the loss of p62 from grade I carcinoma to grade III. We also wanted to evaluate the expression in tumors presenting lymphovascular invasion, a histological feature to be considered a statistically significant prognostic factor [25], and we observed that of the ten cases, seven of them showed a high expression. From these data, it is impossible to establish a prognostic significance of the expression of p62 associated with the different degrees or the presence of lymphovascular invasion, but only by greatly expanding the case series could we obtain a significant result in this sense.

In general, although the data are preliminary, they seem to agree, in terms of p62 expression levels, with those of female breast cancer. In fact, two studies demonstrated a higher expression of p62 in carcinomas compared to normal breast tissue [19,26].

Few studies on p62 and cancer have been performed in veterinary medicine. A study on canine cutaneous mast cell tumors demonstrated nuclear immunoreactivity in low-grade tumors and cytoplasmic immunoreactivity in high-grade tumors [27]. In our study, positivity was always cytoplasmic.

A preliminary study on simple canine mammary carcinoma was more recently published and a reduction in p62 reactivity was observed as the degree of malignancy increased (from normal tissue to adenoma and poorly differentiated carcinoma) [24].

To date, both in human medicine and in veterinary medicine, the knowledge of the role of p62 in oncogenesis is still insufficient and not exactly delineated, partly due to the multiple activities of this protein, but from what we know so far, we can consider this protein as an independent tumor marker and not an oncogene [28].

However, it has been shown that after oncogenic transformation, p62 is necessary for tumor initiation and tumor progression: the induced formation of Her2- breast cancer and the RAS-induced formation of lung cancer are hindered in p62 knockout animals [29,30]. Furthermore, the tumor does not lose its dependence on p62 but becomes dependent, a phenomenon that is known as the non-oncogenic addiction [31]. According to what has been said, it has been shown that the depletion of p62 suppresses the growth of cancer cells in vitro and of the tumor in vivo [32,33]. Another very interesting aspect is that in normal cells, p62 is not as essential as for neoplastic cells since p62 knockout animals develop normally and show only late obesity [34]. This information is very interesting as it points to p62 as a possible target for the development of anticancer drugs.

Another very interesting study area, in which p62 could be involved, is the relationship established between neoplastic epithelial cells, the inflammatory response, and activated fibroblasts associated with epithelial cancer. Studies on human prostate cancer and breast cancer have suggested important metabolic differences between neoplastic cells and fibroblasts, as neoplastic cells are anabolic with a high expression of p62 while stromal cells are catabolic and lose p62 [35,36]. According to the authors, this metabolism, which we can define as asymmetric, promotes tumorigenesis and tumor progression, therefore p62 would play a key role, although in these studies it is not clearly explained how neoplastic cells reprogram fibroblasts.

## 5. Conclusions

This study confirms the importance of the use of the cat as a translational spontaneous tumor model, especially for feline mammary carcinoma, whose analogies with human breast cancer are wildly acknowledged [37]. This preliminary study represents the first approach in the field of feline oncology, and the observation of a high p62 immunohistochemical expression in feline mammary carcinomas represents the first basis for considering p62 as a possible oncological target. Notably, our data are in agreement with that reported for human breast cancer where p62 is overexpressed in malignant cells.

## Figures and Tables

**Table 1:** Immunoreactivity score for p62 in the 38 cases of feline mammary carcinoma.

p62 Positivity Score				
	Low (1–2)	Intermediate (3–4)	High (≥5)	*p*-Value
Grade I Carcinoma (*n* = 5)	-	-	100% (*n* = 5)	0.0919
Grade II Carcinoma (*n* = 10)	20% (*n* = 2)	10% (*n* = 1)	70% (*n* = 7)	0.0666
Grade III Carcinoma (*n* = 23)	21.7% (*n* = 5)	8.7% (*n* = 2)	69.6% (*n* = 16)	>0.9999

**Table 2:** Immunoreactivity score for p62 in cases with lymphovascular invasion.

Cases with Lymphovascular Invasion and p62 Immunoreactivity				
	Low p62 (1–2)	Intermediate p62 (3–4)	High p62 (≥ 5)	*p*-Value
Carcinoma (*n* = 10)	20% (*n* = 2)	10% (*n* = 1)	70% (*n* = 7)	
Grade II Carcinoma (*n* = 2)	-	-	100% (*n* = 2)	0.5556
Grade III Carcinoma (*n* = 8)	25% (*n* = 2)	12.5% (*n* = 1)	62.5% (*n* = 5)	

## Data Availability

The data presented in this study are available on request from the corresponding author.
